# Time, Irreversibility and Entropy Production in Nonequilibrium Systems

**DOI:** 10.3390/e22080887

**Published:** 2020-08-13

**Authors:** Umberto Lucia, Giulia Grisolia, Alexander L. Kuzemsky

**Affiliations:** 1 Dipartimento Energia “Galileo Ferraris”, Politecnico di Torino, Corso Duca degli Abruzzi 24, 10129 Torino, Italy; giulia.grisolia@polito.it; 2 Bogoliubov Laboratory of Theoretical Physics, Joint Institute for Nuclear Research, 141980 Dubna, Moscow Region, Russia; kuzemsky@theor.jinr.ru

**Keywords:** time and action, nonequilibrium statistical thermodynamics, irreversible processes, entropy production, thermodynamics for processes in finite time, temporal evolution

## Abstract

The aim of this review is to shed light on time and irreversibility, in order to link macroscopic to microscopic approaches to these complicated problems. After a brief summary of the standard notions of thermodynamics, we introduce some considerations about certain fundamental aspects of temporal evolution of out-of-equilibrium systems. Our focus is on the notion of entropy generation as the marked characteristic of irreversible behaviour. The concept of time and the basic aspects of the thermalization of thermal radiation, due to the interaction of thermal radiation with matter, are explored concisely from complementary perspectives. The implications and relevance of time for the phenomenon of thermal radiation and irreversible thermophysics are carefully discussed. The concept of time is treated from a different viewpoint, in order to make it as clear as possible in relation to its different fundamental problems.

## 1. Introduction

Thermodynamics serves diverse important functions for physics, chemistry, biophysics and other sciences. It is the branch of physics that develops the analysis of thermal processes and energy transformations. The genesis of thermodynamics has closely been connected with the description of heat processes. The basic question was to find the real limits on the performance of processes and devices.

During these studies, entropy has generally been recognized as one of the most important quantities in physics and related disciplines. There are various forms of entropy in thermodynamics, statistical physics and information sciences [1,2,3,4]. The thermodynamic properties of macroscopic systems may be derived by introducing the appropriate thermodynamic functions of state (or thermodynamic variables) [5,6,7]. Among them, the macroscopic (thermodynamic) entropy *S* has been introduced in order to describe, and characterize, the thermodynamic reversible processes.

The unit of measurement of the entropy is joules/kelvins (J K${}^{-1}$). The joule unit is related to the heat, the energy flux for temperature differences. Kelvins are related to temperature, a thermodynamic quantity that allows us to express macroscopically the molecular kinetic energy [8,9,10,11].

The entropy change ΔS is a marked characteristic of the change of the state of the system, during its evolution between its initial and final states [2,3]. There are numerous macroscopic and microscopic descriptions in relation to the evolution. In accordance with the laws of thermodynamics, for an adiabatic system, or for a closed system operating on a cycle, reversible evolution is an evolution with constant entropy. There are different approaches to the problem of obtaining an irreversible description of processes [5,6,7]. The phenomenological thermodynamics was developed by considering the asymmetry in time. In an isolated system, entropy can only increase with time. This was derived from the second law [12]. In the thermodynamic approach, the entropy generation (the entropy variation due to irreversibility) may be considered as a measure of the irreversibility (however, with some reservations [2,3,13,14]).

The non-equilibrium statistical thermodynamics aims to describe, in a unifying manner, irreversible phenomena, including nonequilibrium steady states and open systems. The workable statistical-mechanical theory of transport processes, in fluids and solids, should maintain these two aspects [1,2,3]:

- It should contain the realistic model, which properly describes the evolution of a system to the equilibrium state;
- It should provide a suitable method for the calculation of the currents and corresponding fluxes.

It is well known that, in a standard thermodynamic approach [5,6,7], one deals with only a small number of state variables; in order to determine the properties of a uniform equilibrium system. For weak nonequilibrium systems, in nonequilibrium thermodynamics [15,16], the method of dividing the system into smaller pieces (subsystems) is used, with the assumption that each subsystem is in a local equilibrium, or quasi-equilibrium, state [1,15,16]. This assumption allows us to characterize each small subsystem by the limited number of physical thermodynamic variables. In particular, when considering the continuous approximation, each subsystem can be characterized by a measurable value of temperature *T*. It is convenient to consider Tσ; σ is the “entropy production”, defined as the time rate of the entropy, created internally by an irreversible process [1,15,16].

In the standard thermodynamics of irreversible processes [5,6,7,15], Tσ is calculated for various irreversible processes, and it is always found to have the form:

$$T\sigma =\sum_{i}J_{i}X_{i}>0,$$

where $J_{i}$ are the flows of the matter, heat, etc., and $X_{i}$ are the generalized driving forces for vector transport processes or for chemical reactions, etc. $J_{i}$ and $X_{i}$ are linearly related when the system is not too far from equilibrium. Thus:

$$J_{i}=\sum_{i}L_{ij}X_{j},$$

where $L_{ij}$ are called phenomenological transport coefficients. The aim of the nonequilibrium statistical mechanics is to derive the macroscopic thermodynamic laws from microscopic time reversible dynamics.

Great efforts have been directed by numerous authors toward establishing the theory of irreversible processes on a microscopic basis [1,2,3,15,16,17,18,19]. These problems are under intense study from various points of view, in spite of the fact that Newtonian dynamics itself is not free from certain shortcomings [20].

In this context, the analysis of a possible link between the macroscopic approach to the irreversibility and microscopic behaviour of a system has been developed [21]. The consumption of free energy has been highlighted to be the cause of the thermodynamic state of the system far from a stable equilibrium. The related entropy generation is the consequence of the redistribution of energy, momentum, mass and charge. Consequently, the concept of the entropy generation represents the essence of the thermodynamic approach to irreversibility. Therefore, irreversibility emerges from the interaction between systems and their environment. Then, lost work was evaluated in a molecular engine, and analogies with macro (classical) heat engines were made [21]. In order to develop this analogy, an irreversible thermodynamics analysis of macro and molecular cycles was developed. Then, this approach was extended to a quantum heat engine with a (−1/2) spin system. In this way, macro and molecular heat engines were shown to obey the same limitations.

The aim of the present review is to clarify some subtle points in the problem of the interconnection of the macroscopic approach to irreversibility and the microscopic behaviour of the systems, in the context of its temporal evolution. This analysis is based on the recent thorough study [2,3] of the concept of time and on the definition of time [22,23] itself, recently introduced by considering a thermodynamic analysis of the “atomic irreversibility” [24,25,26]. The starting assumption is that time may be defined by means of the entropy production and the entropy rate, both due to irreversibility. Consequently, the possibility to design a kind of thermodynamic clock, by using certain properties of a blackbody, has been introduced. The approach is intended to link together (at least partially) the relativistic physics, quantum physics and, in a way, the irreversible thermodynamics, by means of the time hypothesis.

## 2. Entropy and Time

It should be noted that the problem of the proper interconnection of entropy and time has been discussed by various authors, including Clausius, Kelvin, Planck, etc. Long ago, Franklin [27] discussed this question in his study of a class of irreversible processes, which are permanent or steady and the entropy changes of which are associated solely with energy transformations. He devoted to this problem a special section: “The Relation of Entropy and Time”.

From the other side, during the past few decades, the development of these research works has led to new ways of finding natural bounds on performance under the constraint that the system operates at a non-zero rate, thereby giving more realistic bounds than those derived from reversible processes (e.g., the optimization of the Otto cycle) [28,29].

The reasonable approach to establishing those more realistic criteria was the extension of the concepts of thermodynamic potentials and availability, in order to incorporate the constraints on time, or rate, with the other constraints—such as constant temperature, pressure, or volume—in the construction of the potential. Such extensions of thermodynamic potentials, traditionally functions only of the state variables of the system itself, allowed scientists and engineers to include time or rate. These matters have been formulated in the so-called finite-time thermodynamics [30].

When a system is in a nonequilibrium state, it tends to evolve towards the equilibrium state. This evolution may be approximately understood as a succession of stages: quick, slow and steady [1,15,16]. The processes at these stages are governed by diverse characteristic times: relaxation time, collision time, mean-free-path time, etc. There is a hierarchy of various characteristic times [31]. When we consider a closed system in the state of equilibrium, it means that we observe it during a time period that is longer than its characteristic relaxation time.

For open, strong nonequilibrium systems, the situation is different. For such systems, it is necessary to treat them on the time scale that is of the same order of value comparable to, or even shorter relative to, the relaxation time.

The use of time, as an important parameter in specifying the course of an irreversible process, was mentioned and discussed in numerous publications [1].

Honig [32] reconsidered the problems of the interconnection of time and entropy, by taking into account heat transport across boundaries of an open system connected to a reservoir. In terms of classical thermodynamics, the boundary is crossed by heat, and the ratio of this heat flux to temperature can be defined as a flux of entropy [2]. There are no limitations on the sign of this quantity, and one may say that this flux either contributes towards or drains away the system’s entropy. During a reversible process, only this flux can affect the entropy of the system.

Honig’s approach to time-dependent irreversible processes is somewhat different from the more conventional framework. To approximate the properties of systems away from equilibrium, the introduction of a Taylor series expansion about the equilibrium (or nearly equilibrium) state is often required.

Honig [32] reviewed certain technique for determining the entropy change accompanying some classes of irreversible processes, which involve changes in the state of a system. Honig [32] placed emphasis on the time evolution of irreversible processes rather than on setting up time-dependent differential equations and the optimization of available work. He tersely reviewed the fundamentals, by involving the concept of entropy *S* and other functions of state.

Honig [32] noted that, in irreversible processes, the total entropy change in the system is only partially accounted for by the transfer of heat. For adiabatic processes in isolation, entropy can only increase, and a process that continues until quiescence is reached: at this stage, the entropy reaches its maximum with respect to the applied constraints. This result is in accordance with the thermodynamic well-established principles. The operation of the reservoir, in its interactions with the system, that involve heat exchange, mechanical work and transfer of matter, was considered. The state functions were suitably generalized. A deficit function, commonly known as the entropy dissipation function, was introduced into the consideration. The functions of state, which incorporate irreversible processes, were constituted. These functions can then be used to specify the entropy dissipation function for processes under a variety of operating conditions. Time was introduced as a parameter, in order to specify the corresponding entropy evolution of the system under study. In other words, time *t* was introduced as a parameter, in order to simulate the path that the system follows from the initial configuration to the final configuration. The choice of time variations is, to some extent, arbitrary, but guidance was provided by the constructal law proposed by Bejan [33]. The rate of entropy production, associated with the irreversible transfer of heat and material from the reservoir to the system under the specified conditions, was written in the framework of the present formalism. The approach developed may be considered as an alternative method for specifying the entropy change associated with irreversible evolution. The relation to the standard formulation of irreversible processes was examined and discussed.

It should be emphasized, however, that the deep interconnection of time and irreversibility in Honig’s analysis was not clarified in full measure.

## 3. Thermophysics of Radiative Processes and Time

Radiative processes in matter have been a well-developed subject of studies, since Kirchoff, Jeans and Planck [34,35,36,37,38,39,40]. Explicit forms, for radiation temperature and entropy, were obtained by Max Planck [34]. The thermophysics of nonequilibrium thermal radiation fields was studied by various researchers. There is a diversity of approaches and models; we will mention only a few examples [37,38,39,40].

Surdin and collaborators [37] considered a stochastic version of Wheeler and Feynman’s “absorber” theory of radiation, with a classical “zero-point” fluctuating field, corresponding to the residual interactions of all charged particles. The energy spectrum was derived and found to be proportional to a universal constant, identifiable with Planck’s constant *h*. In the framework of their version of stochastic electrodynamics, it was possible to deduce some results of a typically quantum flavour, such as the existence of a stationary ground-level for the harmonic oscillator. However, the problem of entropy was avoided in this study.

Rueda [38] explored the local aspect of the thermalization of thermal radiation due to the interaction of thermal radiation with matter. It was shown that, for absorption and emission interactions, some well-known phenomenological statements of irreversible thermophysics may be relevant. For scattering interactions, the entirely different picture that emerges was also briefly discussed. The local analysis was based on the concepts of the temperature and entropy of nonequilibrium thermal radiation fields. Rueda [38] concluded that the entropy production density is essentially a non-negative quantity. It was shown also that these concepts are susceptible to a very natural thermodynamic interpretation [41].

The role and influence of temperature were investigated by Fonseca et al. [39]. They studied the emission and absorption of radiation by non-relativistic electrons, within the framework of a Lorentz-breaking electrodynamics in (3 + 1) dimensions. Their starting point was the fact that the Planck-type law acquires extra terms proportional to the violating parameters: for the CPT-odd model, the leading extra terms appear to be linear or quadratic in these violating parameters, and according to the background, the vector is parallel or perpendicular to the photon wave-vector. In the CPT-even case, a linear correction shows up. Besides these deviations in the blackbody spectra, those violations may be also probed through a difference in the photon mean occupation number for the two modes. Their results indicate that such violations are better probed at very low temperatures, where their effects on the thermal spectra are largely enhanced.

Boyer [40] summarized the studies of the blackbody radiation in classical physics in a historical perspective and discussed the various subtle questions. He pointed out that relativistic classical electrodynamics, including classical electromagnetic zero-point radiation, gives the Planck spectrum with zero-point radiation as the blackbody radiation spectrum. In contrast, nonrelativistic mechanics cannot support the idea of zero-point energy; therefore, if nonrelativistic classical statistical mechanics or nonrelativistic mechanical scatterers are invoked for radiation equilibrium, one arrives at only the low-frequency Rayleigh–Jeans part of the spectrum, which involves no zero-point energy and does not include the high-frequency part of the spectrum involving relativistically-invariant classical zero-point radiation. Boyer [40] first discussed the correct understanding of blackbody radiation within relativistic classical physics, and then, he reviewed the historical treatment. Finally, the author pointed out how the presence of Lorentz-invariant classical zero-point radiation and the use of relativistic particle interactions transform the previous historical arguments so as now to give the Planck spectrum including classical zero-point radiation. He concluded that within relativistic classical electromagnetic theory, Planck’s constant *h* appears as the scale of source-free zero-point radiation.

These studies left open the problems of time and temporality for the thermophysics of nonequilibrium thermal radiation fields.

Recently, the analysis of irreversibility, of the electromagnetic interaction with atoms and molecules, has been developed by a thermodynamic approach [42]. In the environment, atoms continuously undergo photon-electron interaction due to the environmental electromagnetic fields. This electromagnetic interaction is strictly related to the continuous and universal thermal nonequilibrium, which causes the randomness of atomic and molecular motion. The entropy generation allows us to describe this interaction [43]; indeed, any change in concentration, temperature, volume or pressure generates a readjustment of the system in opposition to the effects of the applied changes in order to establish a new equilibrium, or stationary state [43,44]. Any readjustment of the state of the system can be realised only by generating fluxes of free energy, which entail any process where the system evolves from one state to another. It is plausible to assume that electromagnetic waves are either absorbed from the surroundings of the system and stored in the form of bound energy or emitted from the system to its surroundings as freely propagating photons [43,45,46,47,48,49,50]. The results of this analysis consist of the introduction of an energy footprint in this atomic electron-photon interaction, which has been proven to be related to the spectroscopic phase shift effect and to the experimental results on the irreversibility of the electromagnetic interaction with atoms and molecules, developed in the late 1960s. This quantum footprint has been suggested to be connected with the “origin of time”. This result [42] can be considered also a possible response to the foundational problem highlighted by Einstein on the interaction between radiation and molecules. Einstein argued that the momentum exchange is not usually taken into account in the study of the energy exchange. The recent result [42] was obtained just introducing the momentum into the quantum analysis.

In addition, the authors underpinned their speculations with the hypothesis that time is the consequence of constructal considerations [33,51,52,53,54,55,56,57] of *H*, which is the Hamiltonian of the photon-atomic electron interaction, i.e., from a macroscopic point of view. Moreover, the definition of time [22,23] has recently been introduced by considering a thermodynamic analysis of the “atomic irreversibility” [24,25,26]. Time was pointed out to be related to the entropy production and the entropy rate. Then, a thermodynamic clock was designed by means of the physical behaviour of a blackbody. The result obtained was an attempt to link together relativity, quantum physics and irreversible thermodynamics, on the time hypothesis. Moreover, the result obtained agrees with the idea introduced by Planck and Einstein that the law of the evolution of a system is precisely the law of the evolution of entropy [58,59].

In this context, it is worth mentioning that the problem of the link between the macroscopic and microscopic approaches to physical phenomena may be of interest in the analysis of various problems. This was discussed lucidly in the work by Berry and Smirnov [60], in the context of the physics of clusters. It was pointed out that macroscopic and microscopic approaches are two complementary tools for studying the complex problems, where both approaches begin to coexist. Indeed, the temporal behaviour of entropy and irreversibility in various complex systems [1,2,3] manifest a variety of complicated conceptual problems, which are now under intense study: the relations between quantum mechanics and classical physics, matter-radiation interaction, the electrodynamics of the Wheeler–Feynman model, nanothermodynamics, etc.

We will touch here mainly the question of whether the combined macro (thermodynamic) and micro (motion and interactions of individual atoms) descriptions might provide us with an enlightening description of temporal behaviour and time itself.

Indeed, at the atomic level, the photons can be absorbed by the atomic or molecule electrons, and an electronic energy transition occurs between energy levels of two atomic stationary states. Then, the photons can be also emitted by the excited electrons when they jump down to the energy level of the original stationary state. During this phenomenon, the electrons seem to follow a reversible energetic path, because they come back to the original stationary state of the low energy level. However, as a consequence of the interaction between the atomic or molecule electron and the photon, a footprint occurs in the atom or molecule. The results obtained in [22,24,25] pointed out that the interaction between a photon and an electron in an atom affects the energy level, both of the electron and of the centre of mass of the atom. Therefore, it was analytically proven that the macroscopic irreversibility is a consequence of the microscopic irreversibility due to the photon-electron interaction or, from a macroscopic point of view, between the electromagnetic waves and the matter. Following the results obtained in the analysis of the thermodynamic analysis of electromagnetic fields [61], this interaction can be expressed in terms of entropy production and the power of the electromagnetic wave (the photon) in terms of the entropy production rate. The ratio between the entropy production and of the entropy production rate may be treated as the time of the generation of the irreversibility. Consequently, it has been suggested that the footprint of the irreversibility of any subset of the Universe, due to the fluxes of energy and mass among them [23,42], holds to the definition of the time interval as [23,42]:

(1) $$\tau =\frac{\Delta S}{\dot{S}},$$

where ΔS is the entropy production due to irreversibility and $\dot{S}$ is the entropy rate due to irreversibility. These two quantities are always positive, so the time interval always grows. If we consider the entropy production and the entropy rate per unit volume, we can modify Equation (1) as follows:

(2) $$\tau =\frac{s}{\dot{s}},$$

where [61]:

(3) $$T_{0}\;\dot{s}=\frac{1}{2}\epsilon_{0}cE_{el}^{2}+\frac{1}{2\mu_{0}}cB_{m}^{2},$$

where $E_{el}$ is the electric field, $B_{m}$ is the magnetic field, *c* is the velocity of light, $\epsilon_{0}$ is the electric permittivity in a vacuum, $\mu_{0}$ is the magnetic permeability in a vacuum, *A* is the area of the border of the thermodynamic control volume and $T_{0}$ is the environmental temperature. The entropy production has been obtained by the analysis of irreversibility [62,63,64] of the interaction between a photon and an atomic electron [23,42].

Here, we summarise the bases of the approach. The photon comes into the atomic electron, which jumps from the ground state into an excited energy state, and then, it jumps down to the fundamental state, with the emission of a new photon. The quantum state function, after this interaction, the solution of the Schrödinger equation, can be obtained by the usual quantum mechanical approach, by introducing [22,24,25,47,65]:

(4) $$\psi_{f}\left(r,R\right)=\phi \left(r\right)\;\vartheta_{f}\left(R\right)$$

with ϕ the wave function of the electron and $\vartheta_{f}\left(R\right)$ the wave function of the atom centre of mass [22,24,25,43,48]. A quantum thermodynamic approach to this photon-atomic electron interaction allows us to prove that this atomic process leaves the footprint [22,24,25]:

(5) $$E_{ftp}=\Delta E_{ph}=\Delta E_{CM}=\langle \psi \left(r,R\right)\left|H\right|\psi_{f}\left(r,R\right)\rangle =\frac{m_{e}}{M}\;E_{ph}$$

where H is the Hamiltonian of the photon-atomic electron interaction, i.e., from a macroscopic point of view, the interaction between the electromagnetic wave and matter. Then, by introducing the Gouy–Stodola theorem, it is possible to obtain [22,24,25]:

(6) $$T_{0}S=\frac{m_{e}}{M}\;E_{ph}.$$

Then, considering that for Einstein, any definition of time can only be referred to a clock [66] and following the results of Planck and Einstein [67,68] in relation to the study of the blackbody radiation, the periodic time of oscillation τ for the blackbody radiation, the definition of the time interval was referred to the measurable temperature of a blackbody [22], considering that $h\nu \approx 2.82\;k_{B}T$:

(7) $$\tau =\frac{1}{\nu }=\frac{h}{2.82\;k_{B}}\;\frac{1}{T}\approx \frac{1.70\times 10^{-11}[K\;s]}{T},$$

which highlights the relation between a clock and the temperature. Therefore, the relevant expression for time may be written as [22]:

(8) $$t=n\;\tau =n\cdot \frac{1.70\times 10^{-11}[K\;s]}{T}\approx n\cdot \frac{1.70\times 10^{-11}[K\;s]}{T},$$

where n∈N. We wish to highlight that the relation on the blackbody temperature is related to the peak of the spectrum. Of course, any other reference frequency of the spectrum could be used, but here, we are introducing a clock, a way to measure the time interval (1). With this aim, we chose the peak of the spectrum as the reference for this measurement, because, for this reference, the relation between the temperature and wavelength of the electromagnetic radiation is well known (Wien law).

Consequently, some considerations may be conjectured:

- Time may be considered as a discrete quantity, as pointed out also by Riek [69];
- Time is in a way the result of the irreversibility in the Universe; thus, reversible clocks cannot be realised;
- Locally, entropy can decrease, but entropy generation (due to irreversibility) must always increase, with the consequence that time can only increase, as suggested by the arrow of time;
- Our results are in complete accordance with the ones of Briggs; indeed, only macroscopic classic clocks can be obtained [70].

Now, we wish to link these last results with the ones previously introduced [3]. Indeed, we can point out that:

- Time may be thought of as a manifestation of cyclic processes with ν frequency of the electromagnetic radiation from a blackbody or, in an equivalent way, of the interaction of the electromagnetic wave and the matter;
- The Second Law of Thermodynamics cannot be fully considered only from a mechanical point of view, because there may exist some irreversible phenomena, which may influence or manifest in interaction processes.

Lastly, for the relation between time and action, we need to develop some considerations. Let us consider phase space [71] for the open system, i.e., the set $\Omega =\{\left(x_{i},p_{i}\right),I\in \left[1,N\right]\}$, together with a set Π whose elements π are called process generators. The pair $\Omega_{PA}=\left(\Omega ,\Pi \right)$ is named a system with perfect accessibility. A process in $\Omega_{PA}$ is a pair (π,{(p,x)}). A thermodynamic system is a system with perfect accessibility with two actions, the work *W* and the heat *Q*, exchanged by the system during any process. The set of all these stationary states of a system $\Omega_{PA}$ is called a non-equilibrium ensemble. In this phase space, the entropy density rate $\rho_{S}$ can be introduced together with the Lagrangian density per unit time and temperature $\rho_{L}$, the power density per unit temperature $\rho_{\pi }$ and the dissipation function ϕ. Therefore, considering that [72]:

(9) $$\rho_{L}=\rho_{S}-\rho_{\pi }-\phi$$

with $\rho_{S}-\rho_{\pi }=2\phi$ [72], it was proven that [73]:

(10) $$\rho_{L}=\phi$$

Consequently, the thermodynamic Lagrangian results [45,73],

(11) $$L=\int_{t}dt\int_{T}dT\int_{V}\rho_{L}\;dV=\int_{t}dt\int_{T}dT\int_{V}\phi \;dV=W_{\lambda }$$

Following Gouy and Stodola [74,75,76,77,78], the work lost due to irreversibility can be written as a function of the entropy generation as $W_{\lambda }=-T_{0}S_{g}$. Consequently, the Lagrangian results [45,73]:

(12) $$L=-T_{0}\;S_{g}$$

An analytical thermodynamic approach to this subject allowed us to obtain the mathematical consequences on the behaviour of the entropy generation [45], which seems to maybe have a purely mechanical interpretation in the phase space. Now, introducing the definition of action [45,73,79]:

(13) $$A=\int_{0}^{\tau }L\;dt=-T_{a}\;\int_{0}^{\tau }S_{g}\;dt$$

it is possible to highlight the simple workable relation between the action and the entropy generation. It is clear that the entropy generation of the Gouy–Stodola theorem is no more than the entropy production, because they are the entropy variation related to irreversibility [45,72,73].

## 4. Results and Conclusions

We may summarize the intentions and results of the present study in the following words.

A recent thorough and detailed analysis of time [2,3] pointed out that there are fundamental open questions related to the nature of time and its relation to irreversibility. In particular, the following has been highlighted:

- The dimensional analysis, as also developed by Maupertuis in Analytical Mechanics [80,81,82], yields the relation:
  (14) $$\left[\tau \right]=\frac{\left[A_{0}\right]}{\left[E\right]}$$
  where τ is the time interval, $A_{0}$ is the action, *E* is the energy and [*] means the dimension of the quantity “*”; so, the relation (14) manifests that there is an inherent connection between time and action, as has recently been underlined [83]; indeed, a classical physical system can be described by a set of canonical coordinates (p,q), where *q* denotes the position and *p* denotes the momentum of a particle in the phase space, while $\dot{q}=p/m$. The Hamiltonian $H\left(p,q\right)=p^{2}/2m+V\left(q\right)$ of such a system corresponds to its total energy, where V(q) is the potential. The Lagrangian of such a system results $L\left(p,q\right)=p^{2}/2m-V\left(q\right)$. Maupertuis introduced the following definition of action [82]
  (15) A=∫pdq=∫(L+H)dt
  Now, we follow the approach developed by Landau on the theory of Maupertuis [82]. Therefore, we consider the variation of A [82,84,85]:
  (16) $$\delta A=\delta \int_{t_{1}}^{t_{2}}dtL+H\;\delta \tau +\tau \;\delta H$$
  where $\tau =t_{2}-t_{1}$ is the time, constant, spent by the system along a reversible path connecting two equilibrium states [82]. For a conservative system, the Hamiltonian H(p,q) is conserved in time, so Hδτ+τδH=0, obtaining [82]:
  (17) $$\delta A=\delta \int_{t_{1}}^{t_{2}}dtL=\delta A_{0}$$
  Considering the Hamilton–Jacobi equation in the approach of Maupertuis [82]:
  (18) $$\frac{\partial A_{0}}{\partial t}+H=0$$
  Landau proved [82]:
  (19) $$\frac{\partial A_{0}}{\partial H}=\tau$$
  which is just the relation (14). In this context, some research works have been developed. In particular, to improve the study of space-time, a fundamental and general relation between the entropy variation and the action has been pointed out [58]. Moreover, recently, the link between the principle of least-action, in its original dissipative form, and the arrow of time and the path-dependence, characteristics of the universal evolution, has been pointed out [86,87,88]. All these conclusions confirm our conjectures;
- Time in a way is a manifestation of a cyclic process [3]
  (20) $$t=\frac{1}{\nu }$$
  where ν is a frequency;
- The Second Law of Thermodynamics cannot be considered in full measure from a mechanical point of view only [2,3], so one may suppose that there exists a specific link between irreversible processes and time.

However, these statements are in part conjectures that require a more detailed and thorough study. Nevertheless, we believe that the theses formulated above will be of use and will serve as a stimulus for further research in this direction.

Last, in this review, we also introduced a more realistic numerical evaluation of the time interval in the relation (5), improving our previous results [22].
